# Multifaced Anticancer Potential of Doxorubicin: Spotlight on Breast Cancer

**DOI:** 10.54457/dr.202402015

**Published:** 2025-01-17

**Authors:** Laxita Swain, Biswajit Das, Natalia Baran

**Affiliations:** 1 Department of Biotechnology, MITS School of Biotechnology, 2(P), Infocity, Patia, Chandaka Industrial Estate, Bhubaneswar, Odisha 751024, India; 2 Department of Molecular, Cell and Developmental Biology, University of California, 1156 High Street, Santa Cruz 95064, USA; 3 Department of Leukemia, The University of Texas MD Anderson Cancer Center, 1515 Holcombe Blvd, Houston 77030, USA; 4 Department of Experimental Hematology, Institute of Hematology and Transfusion Medicine, Chocimska 5, Warsaw 00795, Poland

**Keywords:** Anticancer mechanism, Doxorubicin, Breast cancer, Combination therapy, Nanoformulations, Limitations

## Abstract

Breast cancer is a leading cause of death among women worldwide due to its aggressive nature, early metastasis, and resistance to standard chemotherapy. Doxorubicin (DOX) is a potent anticancer drug and remains one of the most effective treatments for breast cancer. This review delves into the diverse anticancer attributes of DOX, encompassing its ability to induce DNA damage, provoke the production of reactive oxygen species, facilitate various mechanisms of cell death, and promote or enhance an anti-tumor immune response. Through an analysis of both monotherapy and combination therapy approaches, this review underscores the immense significance of DOX in contemporary breast cancer treatment. It also delves into the limitations of DOX-based therapies and provides insights into future perspectives for research and development in this field.

## Introduction

Breast cancer poses a worldwide challenge as it is the most frequently detected cancer in women. In 2020, an estimated 2.3 million new cases and more than 685,000 reported deaths were attributed to this disease^[1]^. Per a 2014 American Cancer Statistical Research Report, approximately 232,700 women were diagnosed with breast cancer, representing 29% of all female cancer cases, the most significant proportion among women diagnosed with malignant tumors^[2]^. Unfortunately, 40,000 women died of breast cancer that year, representing 15% of cancer-related deaths and ranking as the second-highest mortality rate among women with cancer. The widespread prevalence of breast cancer underscores the urgent requirement for comprehensive disease management strategies on a global scale^[3]^. The occurrence of breast cancer is higher in high-income nations (571 cases per 100,000 people) compared to low-income regions (95 cases per 10,000 people), indicating its correlation with globalization. Breast cancer is commonly recognized as a spectrum of diseases due to the presence of diverse biological subtypes that manifest distinct molecular profiles and clinicopathological features^[4]^. Apart from histological classifications, gene expression profiling has identified different molecular subtypes of breast cancer, such as receptor-positive types (luminal A, luminal B, normal-like, and HER-2 positive) and receptor-negative types (triple-negative breast cancer [TNBC] or basal-like)^[5]^. TNBC, accounting for about 15% to 20% of all cases of invasive breast cancer, is distinguished by the absence of estrogen receptor (ER), progesterone receptor (PR), and human epidermal growth factor receptor 2 (HER-2) expression on the tumor cell membrane^[6]^.

Several treatment options exist for breast cancer, including surgery, radiation therapy, chemotherapy, hormone therapy, and immunotherapy^[7]^. Chemotherapeutic drugs are categorized based on how they work and their chemical structure^[8]^. These categories include alkylating agents, topoisomerase inhibitors, antimetabolites, mitotic spindle inhibitors, and anthracyclines^[8,9]^.

The anthracycline doxorubicin (DOX) interacts with topoisomerase II, prevents the re-ligation of the ds-DNA breaks, intercalates DNA, induces reactive oxygen species (ROS), has DNA adduct formation properties, and finally works as a mitocan^[10]^. DOX, in addition to other anthracyclines such as daunorubicin, idarubicin, and epirubicin, is commonly employed for the treatment of solid tumors in both adults and children, encompassing soft tissue and bone sarcomas and breast, bladder, ovary, and thyroid cancers^[10]^. Furthermore, DOX is used for the management of Hodgkin lymphoma, small-cell lung cancer, acute lymphoblastic leukemia, and acute myeloblastic leukemia (AML)^[10]^. The FDA has approved the use of the liposomal form of DOX to treat ovarian cancer in patients who have not responded to platinum-based chemotherapy^[11]^. It is also approved for the treatment of AIDS-related Kaposi sarcoma and multiple myeloma^[11]^. Numerous strategies have been pursued to mitigate the adverse effects of DOX, such as employing compounds with antioxidant and/or antiapoptotic properties, devising effective delivery mechanisms, exploring prodrugs, and synthesizing analogues of DOX^[12–19]^. Despite its associated side effects, DOX remains extensively used in cancer treatment, particularly in novel liposomal formulations or drug combinations^[12–19]^.

In this review, we explore recent findings regarding DOX’s mechanism of action, encompassing its involvement in DNA damage, generation of ROS, and induction of apoptosis, autophagy, senescence, ferroptosis, and pyroptosis. Additionally, we delve into the emerging role of this anthracycline drug in modulating tumor metabolism and the immune system and enhancing the antitumor immune response. Furthermore, we discuss the effects of DOX, whether administered as monotherapy or in combination with other treatments, on breast cancer treatment.

## Anticancer mechanism of DOX

### Damage to DNA

#### DOX–DNA intercalation and adduct formation

**Fig. 1.**
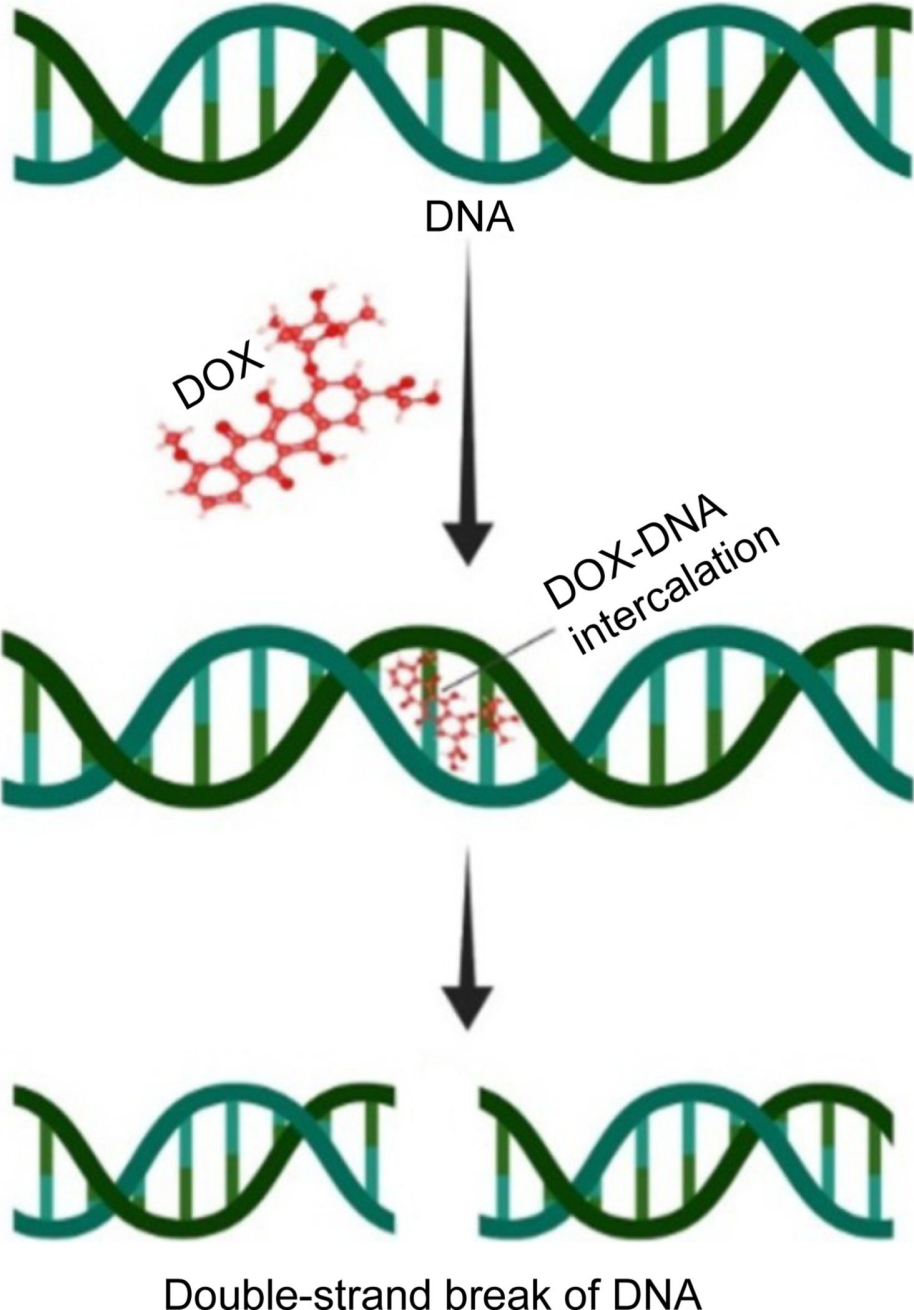
DOX treatment causes DNA damage by the forming DOX-DNA adducts.

DOX, similar to its counterparts in the anthracycline family, interposes within DNA by establishing hydrogen bonds with guanine bases located in neighboring GC base pairs^[20]^. The effect of DOX on cancer cells may be attributed to a suggested mechanism by which DOX’s insertion into DNA causes the molecule to unwind, resulting in positive supercoiling of the DNA helix (Fig. 1)^[20]^. DOX enhances the turnover of nucleosomes adjacent to active gene promoters by intercalating into DNA, leading to alterations in DNA topology^[20]^. The unwinding of DNA triggered by DOX intercalation potentially generates substantial positive torsional stress, thereby destabilizing nucleosomes^[21]^. The development of DOX-DNA adducts can potentially trigger the DNA damage response (DDR) pathway, regardless of the drug's effect on topoisomerase II^[21]^. Notably, these adducts can be identified at drug dosages relevant to clinical practice, indicating their formation during chemotherapy administration.

#### Trapping of topoisomerase

Topoisomerases play a crucial role in DNA replication and transcription by ensuring the integrity of DNA structure^[22]^. They aid in unwinding supercoiled DNA that arises during these processes, introducing either single- or double-strand breaks (SSBs or DSBs) to facilitate template accessibility^[23]^. Primarily DOX inhibits topoisomerase II, but it can also impede the function of topoisomerase I, intensifying its destructive effect on cancer cells. When DOX is administered, it triggers the increase in the expression of genes involved in the DDR pathway, which is linked to the creation of SSBs and DSBs in DNA^[20]^.

Ataxia telangiectasia and Rad3-related (ATR) protein is recognized as the mammalian equivalent of the mitotic entry checkpoint protein 1, commonly called Mec1^[24]^. Mec1 is the key kinase that coordinates DNA damage checkpoints in budding yeast^[24]^. ATR responds to various genotoxic stresses resulting from ultraviolet radiation, DNA polymerase inhibitors, or topoisomerase inhibitors^[24]^. All these stress inducers share the common feature of inducing a pause or delay in the progress of polymerases at DNA replication forks^[24]^. ATR identifies single-stranded DNA formed at stalled replication forks due to MCM2–7 helicase activity. Upon activation through autophosphorylation following DNA damage, ATR kinase is associated with replication protein A/ATR-interacting protein (RPA-ATRIP) complexes on single-stranded DNA, a process facilitated by TOPBP1^[25,26]^. Other factors, such as the MRN complex and RHINO, might aid in recruiting TOPBP1 to activate ATR^[24]^. The ETAA1 protein possesses an ATR-activating domain, and mutations in ETAA1 are viable, unlike TOPBP1 mutations, which result in embryonic lethality in mice^[27]^.

Poly (ADP-ribose) polymerase 1 (PARP1) is known for detecting SSBs in DNA. It collaborates with other biomolecules such as tyrosyl-DNA phosphodiesterase 1 (TDP-1), X-ray repair cross-complementing group-1 (XRCC1), and polynucleotide kinase phosphatase (PNKP) to proficiently rectify irregular DNA termini at single-strand break (SSB) loci^[28]^. For instance, TDP1 changes 3'-phosphotyrosyl bonds to 3'-OH ends, while PNKP eliminates phosphate groups from the 3'-end and facilitates the 5'-hydroxyl groups’ phosphorylation, restoring normal DNA strand ends^[28]^. PARP1 is a crucial coordinator in the SSB repair pathway^[25]^. Within this pathway, PARP1 assumes a critical function in recognizing and transducing DNA damage signals, mediating the conjugation of poly (ADP-ribose) chains to diverse amino acid residues on adaptor proteins and histones located at DNA lesion sites^[25]^. This mechanism facilitates the recruitment of DNA repair factors to the chromatin, encompassing XRCC1, TP53, DNA-dependent protein kinase catalytic subunit (DNA-PKcs), KU70/80, and DNA ligase III^[25,29]^.

When DSBs are detected, cells swiftly mobilize a variety of proteins to the damaged site, creating consolidated multi-subunit formations referred to as foci^[30]^. The MRN complex, consisting of MRE11, RAD50, and NBS1, is pivotal in detecting and managing DSBs^[30]^. MRE11 functions as both a DNA endonuclease and exonuclease, aiding in removing irregular DNA formations, with RAD50 and NBS1 augmenting its capabilities^[30]^. Accumulation of the MRN complex at the site of damage attracts ATM, which becomes activated through monomerization in reaction to DNA damage^[30]^. Upon activation, ATM phosphorylates nearby H2A histone family member X (H2AX) histones, forming γ-H2AX foci, and these foci act as docking sites for MDC1^[25]^. The colocalization of MDC1 enhances the signal by inducing further phosphorylation of H2AX and ATM accumulation at the damage site^[25]^.

DNA-PKcs, part of the PIKK family, has an important function in the DDR system, alongside ATR and ATM kinases^[31,32]^. While ATM mainly facilitates DNA repair through homologous recombination (HR), DNA-PKcs facilitates repair through the non-homologous end joining (NHEJ) pathway^[31,32]^. This process involves the recognition of DSBs by KU70/80 proteins and developing repair complexes by DNA, Ku70/80, and DNA-PKcs. Additionally, DNA-PKcs helps address challenges associated with DNA replication stresses by phosphorylating the ATR-activating RPA32 protein at Ser-4 and −8, thereby activating replication checkpoints^[31,32]^. This function underscores DNA-PKcs’s involvement in protecting genomic integrity during DNA replication checkpoints, as explained below^[31,32]^.

**Fig. 2.**
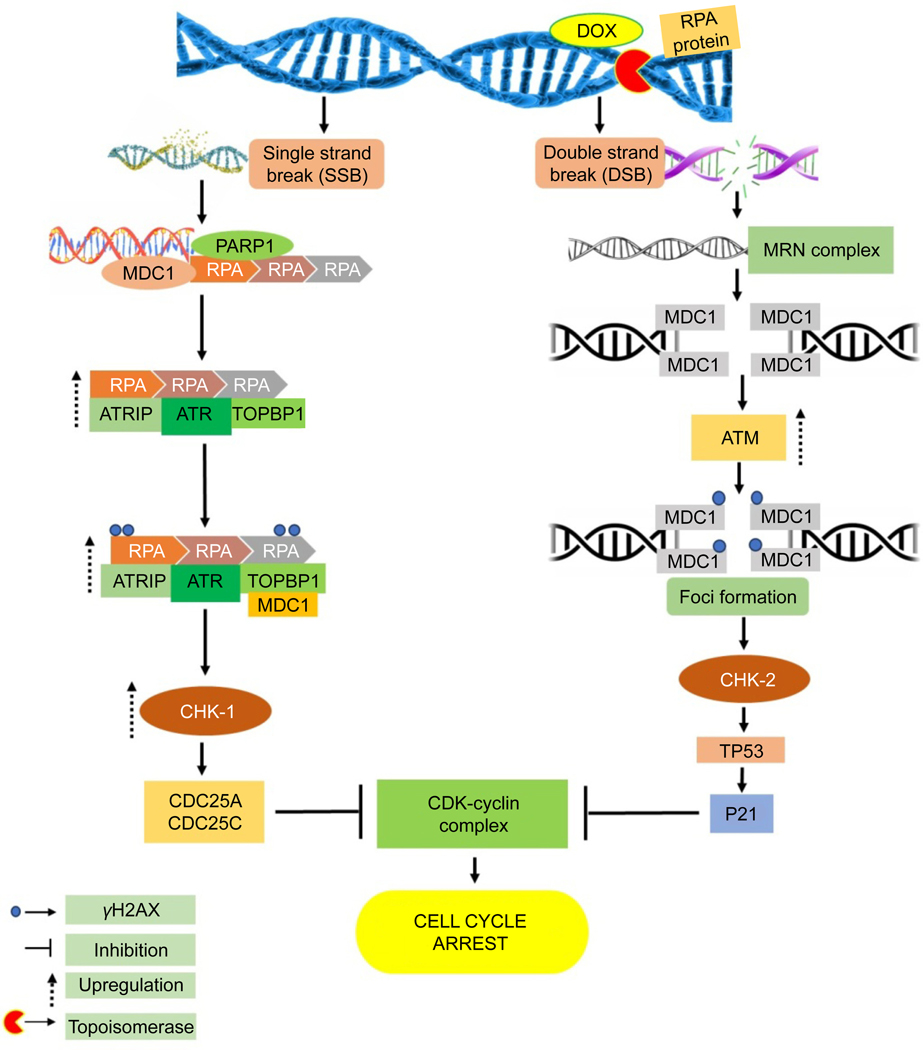
DDR pathway activation occurs due to DOX’s trapping of topoisomerase enzymes, leading to the formation of SSBs or DSBs and, finally, cell cycle arrest followed by cell death^[35]^.

The ATM and ATR protein kinases phosphorylate checkpoint kinases CHK1 and CHK2 to control the DDR. In turn, CHK1 and CHK2 phosphorylate M-phase inducer phosphatases cell division cycle 25 and 26 (CDC25A and CDC25C)^[33,34]^. These regulate the cell cycle by dephosphorylating cyclin–cyclin-dependent kinase (CDK) complexes^[33,34]^. Cell cycle activation is promoted by CDK4 and CDK6 through inactivation of retinoblastoma protein (pRB), while CDK1/CDK2/cyclin A complexes are crucial for progression through the S phase. Phosphorylated CDKs are involved in cell cycle arrest^[33,34]^. Additionally, CDKs phosphorylate upstream elements such as RPA, ATRIP, MDC1, NBS1, ATM, and CHK1^[33,34]^. CHK2, on the other hand, phosphorylates TP53, which modulates the expression of pro-apoptotic genes (e.g., BAX) and inhibitors of anti-apoptotic proteins (e.g., NOXA, PUMA, P21). This process influences apoptosis or cell cycle arrest (Fig. 2) ^[33,34]^.

DOX treatment results in the activation of ATM, which leads to the phosphorylation of NBS1, CHK1, and CHK2 through ATM autophosphorylation on Ser-1981^[35]^. While ascorbic acid does not affect doxorubicin (DOX)-induced phosphorylation of the tumor suppressor protein p53, it implies that it doesnť block this specific DNA damage response^[35]^. Additionally, N-acetyl-cysteine has significantly reduced the phosphorylation levels of several key proteins involved in the DNA damage response, including TP53, H2AX, NBS1, CHK1, and CHK2^[35,36]^. This reduction indicates that hydroxyl radicals, which are reactive oxygen species, may play a crucial role in activating the ATM pathway in response to DNA damage^[35,36]^. The findings suggest that targeting hydroxyl radicals or the ATM pathway could be a potential therapeutic strategy to enhance the efficacy of DOX treatment while mitigating the effects of oxidative stress induced by chemotherapeutics agents^[35,36]^. Activation of CHK2 in cells treated with DOX may proceed regardless of ATM or ATR involvement^[36]^. In acute lymphoblastic leukemia, DOX triggers G2/M cell cycle arrest through the activation of ATR-CHK1, and when ATR-CHK1 is inhibited, synergistic cytotoxic effects are observed^[36]^. Blocking components of the DDR pathway (e.g., CHK1/2, PARP1, ATM, ATR, and DNA-PKcs), enhances the susceptibility of cancer cells to DOX^[37,38]^. DOX-induced suppression of topoisomerase II causes cell cycle arrest at the G1 and G2 phases, subsequently inducing apoptosis^[39]^.

### Apoptosis–ROS interactions

**Fig. 3.**
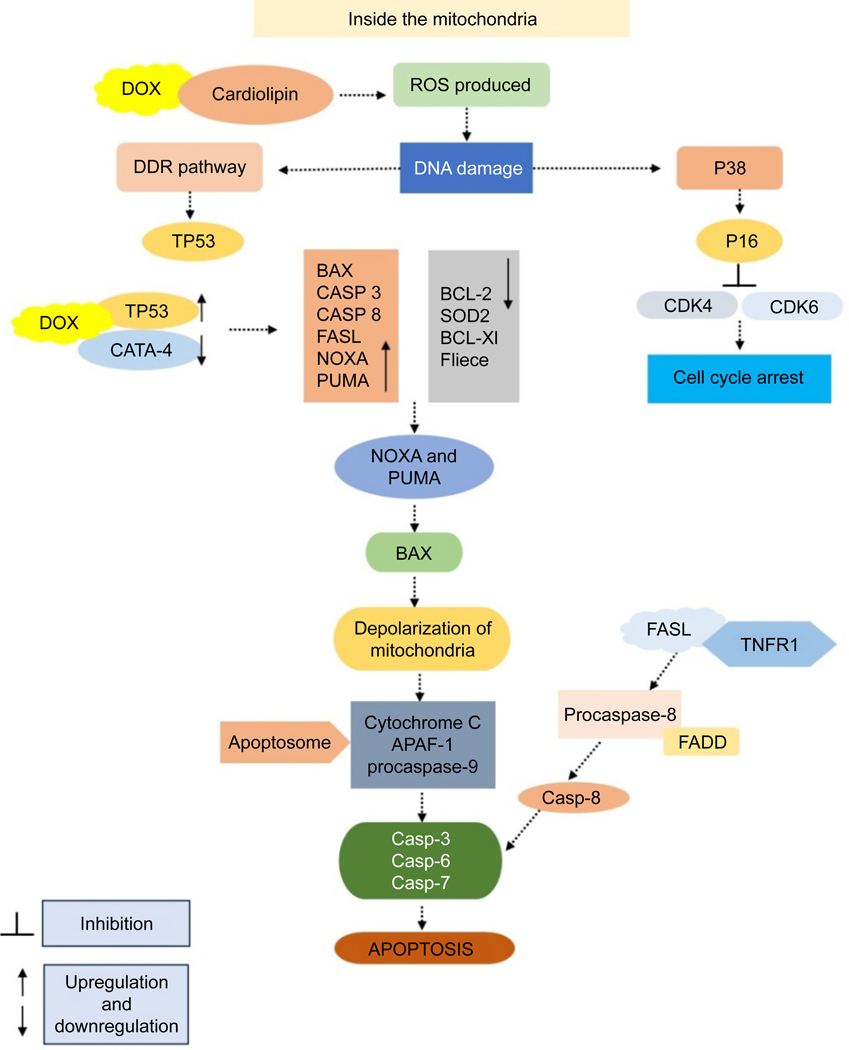
DOX promotes the generation of ROS and programmed cell death by interacting with cardiolipin. This interaction leads to elevated ROS levels, causing harm to both nuclear and mitochondrial DNA. Moreover, DOX stimulates the extrinsic apoptosis pathway by enhancing the expression of FASL, binding to death receptors like TNFR1 and FAS, and activating CASP8 and effector caspases.

ROS can be generated in organisms that use oxygen for energy production through processes such as the electron transport chain, catabolic oxidase activity, and peroxisome metabolism^[40]^. Under normal conditions, ROS are cellular messengers in redox signaling processes when kept at moderate levels^[40]^. However, overproduction of ROS can harm DNA by the action of radicals on DNA bases and its sugar-phosphate backbone^[41]^. Failure to repair the damage may bring about apoptosis, cell cycle arrest, and senescence^[42–45]^. DOX attaches directly to cardiolipin located on the inner mitochondrial membrane., leading to ROS production^[46]^. Increased levels of ROS cause significant damage to mitochondria, resulting in cell apoptosis^[47]^. ROS originating from mitochondria and calcium activate both intrinsic and extrinsic apoptosis pathways induced by DOX in cardiac cells^[47]^. This mechanism entails NFAT-mediated enhancement of FAS antigen ligand (FASL) expression and suppression of FLICE/caspase-8 inhibitory protein (FLIP)^[47]^. In cardiomyocytes derived from induced pluripotent stem cells treated with DOX, there was an increase in death ligands, including tumor necrosis factor receptor 1 (TNFR1), FAS, and death receptor 5 (DR5), which contributed to enhanced apoptosis^[48]^. Moreover, apoptosis was intensified by TNF-related apoptosis-inducing ligand (TRAIL)^[48,49]^. The apoptotic mechanisms induced by DOX and the role of ROS in apoptotic signaling were investigated in osteosarcoma cells. DOX stimulates the production of ROS, which initiates mitochondrial membrane depolarization, cytochrome c release, and caspase-3 activation, ultimately resulting in apoptosis. This process entails elevated levels of BAX and reduced levels of BCL-2 protein^[35]^. Catalase inhibits ROS production, cytochrome c release, caspase-3 cleavage, and apoptosis, underscoring the involvement of ROS in DOX-induced cancer cell death (Fig. 3)^[35]^. The findings indicate that ROS acts as signaling molecules for DOX-induced cell death, even without TP53^[35]^.

In the MCF-7 breast cancer cell line, DOX treatment leads to increased BAX expression, elevated levels of caspase-8 and caspase-3, and decreased BCL-2 expression^[50]^. Additionally, DOX treatment enhances hydrogen peroxide production, reducing NF-κB expression^[50]^. Conversely, in MDA-MB-231 breast cancer cells, heightened SOD2 expression reduces hydrogen peroxide levels and further diminishes NF-κB protein expression^[50]^. This indicates that reduced enzyme activity, including SOD2 and catalases, plays a role in the elevated levels of superoxide observed during DOX administration^[50,51]^. In HaCaT keratinocytes, DOX induces superoxide generation without impacting the levels of hydrogen peroxide, hydroxyl, or peroxyl radicals^[50,51]^. Furthermore, the reduced antiapoptotic activity of the BCL-2 might arise from its ubiquitination followed by proteasomal degradation^[50,51]^. However, the suppression of GATA binding protein 4 (GATA4) expression could lead to a reduction in the antiapoptotic B-cell lymphoma-extra-large (BCL-xL) protein^[51]^. DOX also stimulates the excessive production of ceramide, contributing to ROS generation, DNA damage, and apoptosis^[51]^. Ceramides initiate the release of proapoptotic proteins from mitochondria by forming extensive protein-permeable channels^[51]^. These channels release proapoptotic proteins, including cytochrome c, apoptosis-inducing factor (AIF), procaspases, heat shock proteins, the secondary mitochondria-derived activator of caspases (SMAC)/direct inhibitor of apoptosis-binding protein with low pI (DIABLO), and endonuclease G^[52]^. Mitochondria house the enzymes required for ceramide synthesis and hydrolysis^[52]^. Additionally, evidence suggests an increase in ceramide levels in the mitochondria just before the initiation of apoptosis^[52]^. The process of lipid peroxidation leads to the breakdown of lipid membranes due to oxidative stress^[52]^. This results in the creation of reactive aldehydes, some of which have mutagenic and carcinogenic effects by binding to DNA, forming substances that can cause mutations, and creating links between proteins and DNA, which in turn hinders DNA replication and transcription^[53,54]^. Lipid peroxidation has also been noted after the administration of DOX^[53,54]^.

### Senescence

The concept of cancer cells’ undergoing senescence when treated with chemotherapy is well documented^[55,56]^. As a result, therapy-induced senescence has become a promising approach for fighting cancer with fewer adverse effects^[55,56]^. Research indicates that cancer cell senescence might also have negative effects, as senescent cells can foster an environment that promotes cancer growth. To address this problem, scientists have developed a new class of drugs known as senolytics, which aim to eliminate senescent cells^[57]^. Senescent cells display distinct characteristics such as cessation of cell division, increased production of senescence-associated β-galactosidase, development of heterochromatin foci, shortened telomeres, heightened histone H3K9 methylation, and secretion of various molecules such as chemokines and inflammatory cytokines (e.g., interleukin 1 [IL-1], IL-6, IL-8, MMPs)^[58]^.

### Other categories of cell death

Autophagy is a process in cells activated by lack of nutrients or stressful conditions. It involves breaking down and reusing cellular components to support cell metabolism^[59]^. Autophagy has a two-fold impact on cancer^[59]^. It supports cell survival through catabolic processes, but when autophagic degradation surpasses the cell’s synthesis capacity, it can result in cell death and contribute to necrotic cell death and inflammation in tumors with abnormalities in apoptosis as well as autophagy^[60]^. The loss of autophagy's pro-survival function is recognized to aid in tumor development^[60]^. Autophagy has been seen to obstruct DOX-triggered apoptosis in osteosarcoma and cause resistance to DOX in MCF-7 cells^[61]^. Combining rapamycin with DOX has been shown to enhance cardiac cell viability, reduce ROS generation and apoptosis, and enhance mitochondrial function—both in vitro and in vivo—by stimulating autophagy^[62–64]^. On the other hand, inhibiting autophagy intensified the cytotoxic effects of DOX in breast cancer cells and prostate cancer cells^[62–64]^.

DOX-induced cardiotoxicity involves ferroptosis as an additional mechanism^[35,65,66]^. DOX induces an expansion of the labile iron pool within cells, contributing to its harmful effects^[65,66]^. It disrupts iron homeostasis by deactivating iron regulatory proteins 1 and 2 (IRP1 and IRP2) and influences the expression of genes related to iron metabolism by promoting inactive IRPs' binding with iron-response elements (IREs)^[35,65,66]^. Furthermore, DOX inhibits GPX4, leading to lipid peroxidation, and induces the activation of nuclear factor erythroid 2-related factor 2 (NRF-2), both of which contribute to ferroptosis^[67,68]^.

Pyroptosis, a type of programmed cell death with inflammatory characteristics, has a dual impact on cancer^[69]^. While it can contribute to creating a tumor-promoting environment due to its inflammatory nature, excessive activation of pyroptosis can inhibit tumor cell growth^[70,71]^. Key players in pyroptosis include caspase-1, −4, −5, and −11. Caspase-1 activation leads to the cleaving of precursor forms of IL-18, IL-1β, and gasdermin D (GSDMD) into their active forms^[72]^. Caspase-1 activation occurs through pyroptotic sensors such as the NLR family pyrin domain containing 3 (NLRP3) inflammasome, whereas caspase-4, −5, and −11 are activated through direct interaction with lipopolysaccharides^[67,73]^.

Necrosis commonly occurs when a cell’s ATP levels are depleted, making it less likely for the cell to survive^[74]^. The harmful effects of DOX, such as DNA damage and oxidative stress, can initiate this cell death pathway^[74]^. This is because the ROS produced by DOX results in increased levels of calcium in the mitochondria, ultimately leading to reduced ATP levels by causing the mitochondrial permeability transition pore (MPTP) to open, followed by mitochondrial swelling^[74]^. Many tumors harbor mutations that hinder apoptosis, allowing cells to bypass normal growth cycle checkpoints and continue to proliferate^[74,75]^. Necrosis may be the mechanism by which chemotherapeutic agents such as DOX provoke cell death even when alternative pathways are obstructed^[75]^. Therefore, in situations where apoptosis cannot be initiated, programmed necrosis provides another mechanism of cell death for DNA-damaged proliferating cells, which was initiated by PARP1 and H2AX^[75]^. Programmed necrosis is additionally activated through the TNF and TRAIL death receptor proteins, which inhibit caspase-8 and subsequently stimulate receptor-interacting protein (RIP)^[76]^.

### Immune modulation

DOX enhances the immune system’s capacity to combat cancer^[77]^. It can initiate a specific form of cell death that stimulates the production of interleukins and IFN-γ, while promoting dendritic and T-cell infiltration into tumors^[77]^. When combined with immunotherapy, DOX’s effectiveness is boosted. Both DOX and liposomal DOX show synergetic action with immune checkpoint inhibitors (e.g., monoclonal antibodies against PD-1 and CTLA-4) in mouse models, thus enhancing their antitumor activity^[77]^. Both forms of DOX activate the body's immune response against tumors by attracting CD8-positive T-cells and upregulating CD80 expression in dendritic cells^[77]^. DOX can make cancer cells more responsive to immunotherapy by increasing the expression of activating ligands on cancer cells, making them more susceptible to being killed by immune cells^[77]^.

The death ligand TRAIL binds to TRAIL-R1 and TRAIL-R2 receptors, initiating an apoptotic signal through caspase-8 cleavage^[78]^. The combination of DOX plus TRAIL-targeted therapy sensitizes cancer cells to the apoptotic activity of recombinant TRAIL^[78]^. Furthermore, a sublethal dose of DOX sensitizes cancer cells to NK cells and T-cells by boosting TRAIL receptor signaling^[78]^. This DOX-induced signal boost includes upregulation of TRAIL-R2 expression in cancer cells and reduced expression of cellular FLICE inhibitory protein, a negative regulator of death receptor–mediated apoptosis^[78]^.

## Application of the DOX formulation in breast cancer therapy

DOX can be formulated to more specifically target breast cancer cells, reducing systemic toxicity and minimizing damage to healthy tissues. The formulation of DOX in breast cancer therapy has the potential to improve patients’ treatment outcomes while minimizing adverse effects.

### Liposomal formulation

The negative effects associated with classic DOX therapy necessitated the development of liposomes that could offer comparable efficacy with fewer adverse effects. Liposomal DOX, marketed under the brand names Doxil, Caelyx, or Myocet^[79]^, comprises DOX enclosed within tiny spherical sacs made of phospholipid molecules known as liposomes^[80]^. Liposomal DOX is used to treat breast cancer and other malignancies. It was created based on the idea that liposomes cannot escape the vascular space in areas with tightly packed capillary junctions, such as the heart muscle^[80]^. However, they can exit circulation in tissues and organs lined with cells possessing looser connections, such as tumor cells^[81]^. Consequently, these spheres sustain DOX levels in the bloodstream for extended periods, facilitating greater drug delivery to cancer cells^[81]^.

#### Pegylated liposomal formulation of DOX

Pegylated liposomal DOX (PLD) comprises DOX hydrochloride enclosed within liposomes, with methoxy-polyethylene-glycol (MPEG) attached to the surface^[82]^. PLD evades detection by the mononuclear phagocyte system, resulting in a long-circulating time of up to 66 hours in humans, while the average half-life of PLD is approximately 55 hours^[82]^. PLD has been demonstrated as a crucial treatment option for metastatic breast cancer, both as a standalone therapy and when combined with other treatments, leading to a notable increase in circulation time^[82]^. The drug is encapsulated within the core of the liposome to shield it from metabolism. By covering the liposomes with polyethylene glycol, they can avoid being detected by the immune system, which leads to a longer period of effectiveness^[83]^. Furthermore, specific changes to the surface of the liposomes can allow them to attach to receptors that are abundant on the surface of breast cancer cells, helping minimize the effects on healthy cells^[83]^. Liposomes and micelles have similar properties due to their lipid makeup and are both compatible with the body, break down naturally, and are not harmful or triggering to the immune system^[84]^. When micelles such as Phis-PEG and PLLA-PEG were loaded with DOX, they demonstrated moderate anticancer activity in 4T1 breast cancer cells. PEG-polycaprolactone (PCL)-PEG inhibited the growth of MCF-7 tumor cells when loaded with DOX^[84]^. The loading of DOX into PLLA/PEG also resulted in increased cytotoxicity in MCF-7 cells.

#### Nonpegylated liposomal formulation of DOX

NPLD, a nonpegylated liposomal DOX, introduces an innovative method of drug delivery, marking a significant advancement in cancer treatment^[85]^. It retains the benefits of PLD while avoiding prominent side effects such as hand-foot syndrome. The administration of nonpegylated liposomal DOX offers enhanced safety compared to both regular and liposomal DOX^[85]^. NPLD not only decreases the heart-related side effects of DOX but also helps to alleviate the dose-limiting side effects^[85]^. NPLDs also exhibit prolonged circulation compared to conventional DOX. These advantages are accomplished via a patented specific composition and unique manufacturing process of NPLD’s liposome, giving it the necessary physical and chemical properties^[86]^. Due to the absence of PEG coating, NPLDs do not induce the painful hand-foot syndrome associated with PLDs^[86]^. NPLD combined with cyclophosphamide is presently sanctioned as a first-line treatment for HER2-negative metastatic breast cancer^[87]^. Moreover, it demonstrates significantly lower cardiotoxicity than commonly used anthracyclines and remains efficacious even in patients previously treated with anthracyclines^[87]^.

### Nano-formulation of DOX

Nanotechnology has been instrumental in transforming the way cancer is diagnosed and treated, and the nano-formulation of DOX presents a promising avenue for enhancing the effectiveness and safety of breast cancer treatment by addressing numerous limitations associated with conventional chemotherapy^[88]^. Nanoparticles can be classified into two main groups: inorganic nanoparticles such as gold nanoparticles, quantum dots, iron oxide, and paramagnetic (europium-based); and organic nanoparticles such as dendrimers, micelles, liposomes, ferritin, and others^[88]^.

#### Inorganic nanoparticle formulations

Different nanoparticle systems, such as gold nanoparticles, offer potential solutions to the challenges encountered in DOX-based breast cancer treatment^[79]^. Gold nanoparticles, typically smaller than 150 nm, have a gold core and are considered biologically inert and non-toxic. Due to their biocompatibility, gold nanoparticles have recently attracted interest as potential carriers for delivering anticancer drugs^[89,90]^. Gold nanoparticles loaded with DOX were selectively internalized into MCF-7 cells, leading to enhanced cytotoxic effects explicitly targeting cancer cells^[91]^. Silica nanoparticles (SiNPs) loaded with DOX, with a particle size of 189 nm, demonstrated sustained release characteristics, aiding in maintaining an optimal drug concentration in the bloodstream over an extended period^[92]^.

Modifying the DOX-loaded calcium carbonate (CC) nanoparticles with high-density lipoprotein (HDL) resulted in a slight increase in size with a consistent size distribution and negative surface charge^[93,94]^. These HDLCC-DOX nanoparticles demonstrated improved killing of MCF-7 cells and strong anticancer effects in an in vivo model, leading to reduced tumor growth and adverse effects compared with free and DOX CC-DOX. Those studies established the tumor-targeting ability and improved the safety of HDL/CC/DOX nanoparticles^[93,94]^.

Various carbon-based nanomaterials serve as targeting tools for anticancer agents. These materials include carbon nanotubes, carbon nanofibers, fullerenes, and carbon black^[95]^. In one study, multi-walled carbon nanotubes carrying DOX, folic acid, and estrone-anchored PEG showed targeted anticancer activity toward cancer cells^[95]^. Magnetic nanoparticles (MNPs) direct therapeutic agents to specific locations while reducing side effects by using magnetic fields to manipulate electric fields between nanoparticles and cancer cells, taking advantage of their unique electric properties^[96,97]^.

#### Organic nanoparticles formulation

Polymeric nanoparticles (PNPs) are made from natural components, e.g., chitosan, dextran, polylactic acid, polylactide-coglycolide, or PCL. They range in size from 10 to 1000 nm and can encapsulate drugs^[98]^. PNPs offer benefits such as biocompatibility, biodegradability, and versatility in design. Chitosan, a polysaccharide made of D-glucosamine units, has antibacterial properties without immunogenic or carcinogenic potential^[98]^. Oleic acid was used to prepare oleyl chitosan, which inhibits oncogene promoters in breast cancer and enhances chemotherapy effectiveness^[99]^. DOX-oleyl chitosan showed superior efficacy in suppressing breast cancer cell growth compared to free DOX^[99]^. Hyaluronic acid is associated with cancer progression and metastasis, and breast cancer cells exhibit elevated expression of CD44, the main receptor for hyaluronic acid. Conjugating DOX with N-(2-hydroxypropyl) methacrylamide copolymer displayed promise in targeting breast cancer and combating metastasis^[100]^. Phase I clinical trials have demonstrated its effectiveness in treating drug-resistant breast cancer. Binding this copolymer conjugate with aminoglutethimide, an aromatase inhibitor, enhanced its cytotoxic effects against breast cancer cells^[100]^.

Solid-lipid nanoparticles (SLNs) represent a promising colloidal carrier system for improving the efficacy of anticancer agents^[101]^. By incorporating anticancer agents within colloidal nanoparticles, drug resistance can be overcome, leading to increased drug concentrations within cancer cells, including those of breast cancer^[101]^. Compared to free DOX, DOX-loaded SLNs exhibited significantly higher accumulation in MCF-7/ADR cells^[101]^. Specifically, the relative cellular uptake of DOX -loaded SLNs was 17.1-fold higher at 60 minutes and 21.6-fold higher at 120 minutes compared to the free drug^[102]^. Protein-based nanostructures, such as modified human serum albumin nanoparticles, were employed with an outer coating of polyethylenimine to enhance the therapeutic effectiveness of DOX in breast cancer cells^[102,103]^. Micelles are colloidal nanoparticles or nanocarriers that self-assemble, typically exhibiting an average particle size of 5 to 100 nm^[102,103]^. Incorporating DOX into micelle formulations has been shown to enhance its therapeutic effectiveness, particularly against cancer stem cells in TNBC^[102,103]^. When combined with DOX, dextran-retinoic acid exhibited potent anticancer activity in MCF-7 breast cancer cells^[102,103]^. The synergistic anticancer activity in the HeLa cell line was enhanced by loading DOX into carboxymethyl chitosan^[104]^. Loading DOX into hyaluronic acid resulted in significant inhibition of tumor growth in MCF-7 cells^[105]^. Likewise, incorporating DOX into poly(ε-caprolactone)-polyphosphoester enhanced anticancer activity in MCF-7 cells^[105]^. Combining DOX with Tetronic T1107, Pluronic F127, and TPGS resulted in potent anticancer activity in MDA-MB-231 breast cancer cells^[106]^.

Dendrimers, characterized by their hyperbranched, spherical, and three-dimensional structure, serve as nanocarriers for various anticancer drugs to treat breast cancer^[107]^. Polymeric dendrimers, such as PAMAM dendrimers, are widely used in biomedical applications because of their low toxicity^[107]^. PAMAM dendrimers loaded with DOX exhibit increased cellular uptake and binding affinity in the T47D and BT-549-Luc cell lines^[108]^. Incorporating DOX into Pluronic F68-PAMAM increased antitumor activity in the MCF-7/ADR cells^[109]^. Loading DOX into collagen enhanced its potential anticancer efficacy in the MCF-7 cell line^[110]^, see Table 1.

## DOX monotherapy and combination therapy in breast cancer

### Monotherapy

DOX monotherapy is commonly utilized when it is considered effective for treating a specific type of cancer^[113]^. Apoptosis triggered by DOX was assessed by examining gene and protein expression levels of caspase-3, caspase-8, and caspase-9 in breast cancer cell lines. Besides its direct cytotoxic effects, DOX contributes to cancer cell elimination by activating immune CD8-positive T-cell responses. Anthracyclines like DOX directly eliminate tumor cells and enhance antitumor immunity^[113]^. During the process of cell death, the tumor microenvironment releases cellular contents, including tumor antigens and signals known as damage-associated molecular patterns (DAMPs), which can boost the body’s immune response against the tumor^[113]^. These DAMPs can start an inflammatory reaction, attract immune cells, and assist in identifying tumor cells^[114]^. This process is called immunogenic cell death. DOX has been found to induce immunogenic cell death, leading to the activation of a dendritic cell-mediated, tumor-specific CD8-positive T-cell response^[114]^. Additionally, studies have demonstrated that DOX selectively killed myeloid-derived suppressor cells in the tumor microenvironment, thereby mitigating their immunosuppressive effects, in a breast cancer model^[113]^.

Researchers investigated the use of both photothermal and chemotherapy in MCF-7 breast cancer cells by using DOX-loaded gold nanocages enclosed in thermosensitive liposomes^[115]^. This investigation was conducted in both in vitro and in vivo environments^[115]^. Both MCF7 and SKBR3 breast cancer cells have an overabundance of HER-2 receptors^[115]^. Studies on cell toxicity demonstrated that DOX-loaded liposomal formulations caused more cell death than free DOX in both MCF7 and SKBR3 cells^[116]^. This increased effectiveness is attributed to the rapid uptake of liposomes through endocytosis, which reduces the degradation of sensitive drugs. In contrast, drug solutions enter cells solely through passive diffusion^[116]^.

DOX affects the activity of NF-κB, a transcription factor involved in controlling genes related to cell growth, development, and apoptosis^[117]^. Overactivation of NF-κB is linked to increased cellular functions in various tumors. In different cell lines, DOX can either upregulate or downregulate NF-κB gene expression but decreases NF-κB protein expression^[117]^. Inhibiting NF-κB can enhance apoptosis in cancer cells, including breast cancer when treated with DOX^[117]^. In MCF-7 cells, DOX reduced the expression of the anti-apoptotic Bcl-2 protein and increased oxidative stress by increasing hydrogen peroxide production while decreasing both NF-κB gene and protein expression^[117]^.

**Fig. 4.**
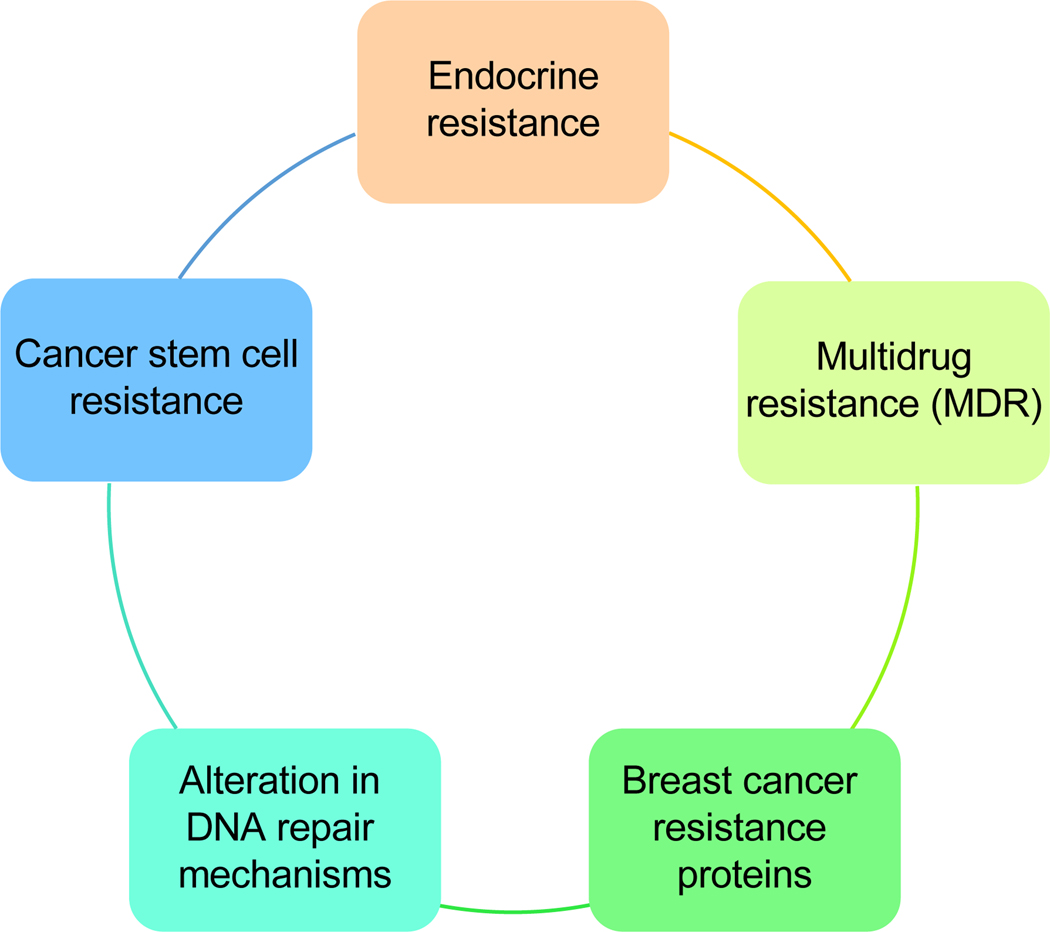
The mechanisms of resistance in breast cancer are complex and multifaceted, often arising due to genetic mutations, tumor heterogeneity, and the microenvironment. These factors can enable cancer cells to evade therapies such as hormonal treatments and chemotherapy, resulting in treatment failure. This figure illustrates the various types of resistance that have developed in breast cancer.

Resistance in breast cancer cells can occur due to several various factors, but one of the main reasons is the development of resistance to chemotherapy drugs^[118]^. The development of resistance can happen through multiple mechanisms (Fig. 4). Dealing with drug resistance in breast cancer necessitates a comprehensive strategy that involves creating fresh treatment approaches that focus on particular resistance mechanisms, using combination therapies, and tailoring treatment based on the tumor’s molecular traits.

### Combination therapy

DOX monotherapy may develop resistance over time. However, administering DOX in combination with other agents can reduce the likelihood of acquired resistance to DOX. This approach depends on diverse mechanisms, such as enhancing efficacy (additive, synergistic, or potentiating effects), diminishing resistance, improving tolerability, and contributing to better treatment outcomes^[119–192]^. An overview of diverse combinatorial approaches, including chemotherapy agents, small molecules, off-label agents, and plant- and marine-derived substances, is summarized in Tables 2–4.

## Limitations of DOX-based therapies

DOX’s therapeutic benefits are tempered by adverse effects on healthy cells, most notably cardiotoxicity. Several significant factors can influence anthracycline cardiotoxicity^[191]^. These include the specific type and total dosage of chemotherapy administered, method and schedule of administration, concurrent use of other medications known to affect the heart, and potential combination with chest radiation therapy^[192]^. Furthermore, specific qualities such as female sex, hypertension, coronary artery disease, obesity, type 2 diabetes, congestive heart failure, previous exposure to anthracyclines or radiation, valve issues, initial left ventricular function, African-American heritage, age extremes, renal dysfunction, and electrolyte imbalances, constitute a group of acknowledged and significant factors. Moreover, genetic predispositions and environmental influences likely affect susceptibility to anthracycline-induced cardiac damage^[192,193]^. The mechanism underlying DOX-induced cardiac toxicity diverges from its antitumor action. It entails heightened oxidative stress, the suppression of cardiac-specific genes, and the initiation of cardiac myocyte apoptosis by DOX^[194,195]^. DOX-induced irreversible cardiomyopathy can manifest within months following treatment cessation, but cases have been documented up to two decades later^[195–197]^. Congestive heart failure is a potential consequence. Among the risk factors for DOX-related congestive heart failure are higher cumulative dose of DOX, extremes of age, concurrent chemotherapy with other cardiotoxic agents, pre-existing left ventricular dysfunction, hypertension, and prior radiation therapy to the mediastinal area^[195–197]^. Patients who experience congestive heart failure after DOX treatment have a 1-year mortality rate of around 50%^[195–197]^. Unfortunately, 10% to 75% of cancer survivors experience chronic cardiovascular complications later in life due to the toxicity of their treatment^[198]^.

Furthermore, approximately 5% of patients treated with anthracyclines exhibit signs of congestive heart failure or experience a notable dose-dependent decrease in left ventricular (LV) function^[199]^. The resulting cardiac impairment from anthracyclines limits cancer treatment^[199]^. Dexrazoxane is the only intervention to mitigate DOX-induced cardiotoxicity, but the FDA restricts its use^[199]^. The most common side effects in patients treated with dexrazoxane include acute nausea and vomiting, stomatitis, gastrointestinal problems, alopecia, neurologic disorders (hallucinations, vertigo, dizziness), cumulative cardiotoxicity, and bone marrow aplasia^[199]^.

The scope of DOX includes healthy tissues with high rates of cell division, such as myeloid and lymphoid tissues, the lining of the gastrointestinal tract, and reproductive organs^[199,200]^. Due to the notable increase in survival rates for individuals with cancer in the past 20 years, there has been a marked uptick in the population of cancer survivors who encounter DOX-related damage to their reproductive systems^[200]^. The DNA-damaging effect of DOX is a primary factor contributing to gonadotoxicity, particularly in female patients. In male gonadotoxicity, there may be additional influence from specific histone eviction, particularly in the case of dimethyl DOX^[201]^.

## Strategies to overcome DOX-induced toxicities

Presently, the primary strategies to mitigate DOX-induced cardiotoxicity involve early detection, limiting the cumulative lifetime dose, extending the duration of intravenous anthracycline infusions, addressing significant cardiovascular disease risk factors (such as smoking cessation, managing metabolic disorders, and promoting physical activity), and implementing both pathogenetic and symptomatic therapies (including beta-blockers, statins, and RAAS inhibitors)^[192]^. When treating patients with DOX-induced cardiotoxicity, it is crucial to conduct risk stratification, enabling the selection of optimal strategies to monitor and manage their cardiovascular disease^[192]^. Several prospective drugs and targets are demonstrating cardioprotective potential. These include thrombopoietin, sestrins, ghrelin, and sirtuins, as well as natural phytocompounds such as resveratrol, flavonoids, vitamin E, and lotusine, in addition to cardioprotective strategies aimed at mitochondria^[192]^. However, further well-designed clinical studies are needed to evaluate the effectiveness of these substances. In addition, low concentrations of carbon monoxide (CO) have been shown to protect against DOX toxicity^[202]^. CO has cardioprotective and anti-tumor effects. It may help reduce injury and inflammation. HBI-002, a liquid drug containing CO, has completed a phase I trial and could be used to limit cardiac damage in cancer patients undergoing anthracycline therapy^[203,204]^. Anthracycline combined with cytarabine is a key part of AML induction therapy, but drug resistance can be a challenge. L-Annamycin, a new anthracycline, has shown promise in overcoming multidrug resistance in both preclinical and clinical studies, with reduced cardiotoxicity compared to standard anthracyclines like DOX^[205,206]^. The encouraging characteristics of L-Annamycin have led to phase I/II clinical trials in Europe and the United States for treating relapsed/refractory AML. L-Annamycin's ability to overcome MDR and its absence of cardiac toxicity presents promising prospects^[206]^. These merits fully support the implementation of a phase II clinical trial aimed at demonstrating L-Annamycin’s efficacy in all patients with relapsed/refractory AML, regardless of mutational status. This ongoing study is being conducted across five sites in Poland and three in Italy^[207]^. Selenomethionine activates GPX4, reducing PUFAs and oxidized lipids. It inhibits DOX-induced ferroptosis through a GPX4-dependent mechanism without compromising chemotherapy’s effectiveness, suggesting it is a potential therapy for preventing DOX-induced cardiotoxicity^[208]^.

## Conclusion

Although DOX is a commonly used and effective chemotherapy for breast cancer, there is still ongoing debate about its specific mechanism of action. While the details are not yet fully understood, it is known that DOX inserts itself into DNA, blocks topoisomerase enzymes, disrupts mitochondrial function, and increases the production of free radicals, which leads to oxidative damage. In addition to initiating the mitochondrial apoptosis pathway, DOX can cause cancer cells to undergo senescence, autophagy, pyroptosis, ferroptosis, or necrosis, with a specific response depending on the drug dosage and the cellular environment. The use of DOX can lead to serious side effects in healthy cells, such as heart problems. Despite these challenges, there are ongoing efforts to improve the safety of DOX. Different forms of DOX, such as liposomal encapsulation and nanoparticles, have been created to make it more effective with fewer side effects. Researchers have also been studying combination chemotherapy with DOX and other anticancer drugs to overcome drug resistance and minimize side effects. This is important because using DOX alone may not effectively stop breast cancer growth due to the uneven distribution of cancer cells in tumors. However, it remains difficult to achieve strong anticancer effects while minimizing harm to healthy tissue because of the unique properties of the drugs involved.

## Future perspective

In the future of breast cancer treatment, DOX is expected to remain important. Scientists are working to ensure that DOX goes directly to the cancer cells without harming healthy cells. They are exploring new methods, such as using nanoparticles and linking DOX to antibodies, to reduce side effects and make the treatment more effective. Researchers are also trying to find ways to predict how well patients will respond to DOX so they can personalize the treatment based on each person’s tumor and characteristics. In the future, scientists will focus on understanding why some tumors do not respond to DOX and will work on strategies to overcome this. They will also try to find ways to protect the heart from potential damage caused by DOX. Overall, the future of DOX treatment for breast cancer depends on using new ways to deliver the drug, personalizing treatment, and using combinations of treatments to make them as effective as possible while causing as few side effects as possible. Understanding how DOX works inside tumor cells is an important area of ongoing research.

## Figures and Tables

**Table 1.** Different formulations of DOX in breast cancer therapy.

Sl. No	Formulation Type	Role in Cancer Therapy	References
1.	Pegylated liposomal formulation of DOX	prolongs presence of DOX in the bloodstream, allows for increased delivery of the drug to cancer cells.	[111]
2.	NPLD	cardiac toxicity associated with DOX and alleviates dose-limiting toxicity linked with liposomal DOX.	[85,86]
3.	Nano-formulation of DOX	boosts effectiveness and efficacy.	[91,92,94,98,100,105,112]

NPLD: Nonpegylated liposomal DOX.

**Table 2.** The Role of DOX in Combination with chemotherapy and small molecule inhibitors for Breast Cancer treatment.

DOX-based Combinations	Function	Breast Cancer Type	References
gemcitabine-incorporated APTA12	Enhances cytotoxicity	1	[132–134]
MK-220	GLUT1 inhibitor, ↓ Akt & ↑ ROS-triggered cytotoxic effects → DNA damage & hindering repair processes, ↑ apoptosis	1, 2	[140]
WZB117	GLUT1 inhibitor, ↓ Akt & ↑ ROS-triggered cytotoxic effects → DNA damage & hindering repair processes, ↑ apoptosis	1, 2	[140]
Organometallic Ruthenium compounds	↓ PI3K/AKT signaling pathway	2	[141]
Paclitaxel	↓ tubulin	2	[148]
Docetaxel	↓ microtubule depolymerization → abnormal mitosis	1, 2	[149]
Gamitrinib	Cytotoxic activity is boosted by stimulation of the CHOP and JNK signaling pathways, activation of proapoptotic proteins, ↑ caspase → cell death	1	[150]
Mitomycin C	Enhances synergy leading to more DNA double-strand breaks.	3	[152]
ATRA	Inhibits cell proliferation.	2	[153]
Dasatinib	Blocks growth, invasion & migration	1,2,4	[158]
Lapatinib	Inhibits ABC transporter expression, including MDR-1 and BCRP, to boost DOX accumulation inside cells.	2	[178]
Apatinib (rivoceranib)	Diminishes proliferation and migration; triggers apoptosis.	1	[185]
Abemaciclib	↑ Expression of cleaved caspase-3, cleaved PARP & Bax	1	[190]

(1) MDA-MB-231, (2) MCF-7, (3) All clinical subtypes, (4) T47D, ATRA- All-trans retinoic acid.

**Table 3.** The Role of DOX in Combination with other agents besides of chemotherapy for Breast Cancer treatment.

DOX-based Combinations	Function	Breast Cancer Type	References
Dihydroartemisinin	Induces PARP cleavage, activates the caspase cascade	2	[119–121]
Metformin	Enhances cancer cell responsiveness to DOX	5	[122,123]
Melatonin	Autophagy-dependent transcriptional reduction of AMPKα1 mRNA, ↑ apoptosis	6	[143]
Noscapine	Suppresses tumor growth via NF-KB deactivation, ↑ apoptosis & angiogenesis limitation	5	[130,131]
Niclosamide	↑ apoptosis, blocks Wnt/β-catenin signaling, cell cycle arrest at G0/G1 phase, ↑ ROS levels→ cell death	3	[135]
Amphotericin B	Apoptosis induction	2	[159,160]
Quinacrine (Mepacrine)	Triggers apoptosis via sub G1 arrest and downregulates Nrf2, Bcl-xl, and cyclin B1.	1	[154]
Hydralazine	↑ apoptosis & ↓ proliferation	2	[163]
Disulfiram	↑ apoptosis & ↓ proliferation	2	[163]
Zoledronic acid	↑ apoptosis	7	[164]
Empagliflozin	Reduction of MDR1 gene expression enhances DOX’s cytotoxic & apoptotic effects	5	[165]
Simvastatin	↑ ROS, upregulating p21, enhancing cytochrome c and caspase-3 expression, and reducing cyclin D1 → ↑ apoptosis.	2	[174]
Sulbactam	Boosts cytotoxicity by hindering the transcription and translation initiation of proteins associated with ABC transporters, inducing apoptosis.	8, 9	[175]
Vitamin D	Modifies F-actin and vimentin structure, diminishing cancer cell survival.	2	[177]
Calcifediol	Inhibits cell growth.	2	[182]
Tramadol	Inhibits cell growth, migration, colony formation & invasion; induces cytotoxicity.	1, 2	[187]
Ozone	Enhances the anti-proliferative & DOX-induced apoptosis	10	[188]

(1) MDA-MB-231, (2) MCF-7, (3) All clinical subtypes, (5) TNBC, (6) MDA MB157, (7) MDA MB 436, (8) MDA MB 468, (9) MB-453, (10) BT474.

**Table 4.** The Role of DOX in Combination with plant-and marine-based substances for Breast Cancer treatment.

DOX-based Combinations	Function	Breast Cancer Type	References
Naringenin	Enhances cancer cell responsiveness to DOX	5	[122,123]
PSP	Apoptosis induction by creating an S-phase trap & boosting the host immune response	1,2,4	[124–126]
Curcumin	Modulates regulatory proteins and signaling pathways	1, 2	[127–129]
Piperlongumine	↓ JAK2-STAT3 pathway hampers cell proliferation & ↑ apoptosis	5	[136–139]
Hesperetin	Cell cycle arrest & ↑ apoptosis	11	[142]
Oleuropein	↓ NF-ΚB and its downstream targets cyclin D1, BCL-2 & survivin.	1	[144]
Renieramycin M	↑ apoptosis by regulating ErbB/PI3K-Akt, integrin & focal adhesion signaling pathways	2	[145]
SHOO3	↑ apoptosis	5	[146]
Genistein	↑ cell cycle arrest & apoptosis.	2	[147]
Oridonin	Apoptosis induction by modulation of Bcl-2/Bax, PARP, caspase-3 & survivin pathways, reducing proliferation, migration, & invasion	1	[151]
Furanodiene	Triggers apoptosis through mitochondria-caspase pathways independent of ROS	12	[155]
Peiminine	Hinders DNA repair by suppressing MAPK signaling pathways	1, 2	[156]
Nitidine chloride	Triggers G2/M cell cycle arrest	1, 2	[157]
Gingerol	↑ active caspase-3 and γH2AX levels, ↓ Cdk-6 cyclin levels	5,13	[161]
Lycium barbarum	Induces cytotoxic effects	1, 2	[162]
Sulforaphane	Suppressing HDAC6 expression induces autophagy	5,8,14	[166]
Lectin	Arrests cells in the S phase and reduces the number of cells in the G0/G1 phase	1, 2	[167]
Tanshinone IIA	Inhibiting the PTEN/AKT pathway reduces expression of efflux ABC transporters, e.g., P-gp, BCRP, and MRP1	2	[168]
Quercetin	↓ tumor necrosis & reduces DOX side effects on non-tumoral cells. ↓ reduces activity of efflux ABC transporters, e.g., P-gp, BCRP, and MRP1.	1, 2	[169–171]
Magnoflorine	Promotes apoptosis via Caspase-3 cleavage, ↓ PI3K/AKT/mTOR pathway, boosts the p38 MAPK pathway	2	[172]
Carotenoids	Causes mitochondrial dysfunction, cell cycle arrest at the G0/G1 phase, activation of caspase cascades	2	[173]
Rhinacanthin-C	Enhances DOX cytotoxicity by blocking MRP2 and P-gp functions.	2	[176]
Grape seed extract	Triggers G1 phase arrest.	8	[179]
Enoxolone (Glycyrrhetinic acid)	Boosts cytotoxicity and apoptosis; disrupts mitochondrial membrane potential by enhancing the mitochondrial-driven apoptosis pathway.	2	[180]
Resveratrol	Suppresses proliferation and triggers apoptosis by inhibiting chronic inflammation and autophagy.	1, 2	[181]
Soursop leaf extract	Induces anticancer property.	2, 4	[183]
Curcumol	Heightens susceptibility to DOX.	1	[184]
Vanillin	Halts cancer cell proliferation, induces apoptosis & suppresses tumor growth.	2	[186]
Cinnamon essential oil	Triggers apoptosis & shows cytotoxic effects	4	[189]

(1) MDA-MB-231, (2) MCF-7, (4) T47D, (5) TNBC, (8) MDA MB 468, (11) HER2, (12) ERα-, (13) 4T1Br4, (14) BT 549, PSP-Polysaccharopeptide.

## Abbreviations

ABC: ATP synthase-binding cassette transporters
ADP: Adenosine diphosphate
DR cells: Doxorubicin resistant cells
AIDS: Acquired Immunodeficiency Syndrome
AIF: apoptosis-inducing factor
Akt: Ak strain transforming
AML: acute myeloid leukemia
ATM: Ataxia-telangiectasia mutated
ATP: Adenosine triphosphate
ATR: Ataxia telangiectasia and Rad3-related
ATRA: All-trans retinoic acid
ATRIP: ATR-Interacting Protein
BAX: BCL-2 associated X
BCL-2: B-cell leukemia/lymphoma 2 protein
BCL-XL: B-cell lymphoma-extra-large
BCRP: Breast Cancer Resistance Protein
CC: calcium carbonate
CD80: Cluster of differentiation 80
CDC25: cell division cycle 25
CDK: Cyclin-dependent kinase
CHK: Checkpoint kinases
CHOP: C/EBP homologous protein
CO: carbon oxide
CTLA-4: cytotoxic T-lymphocyte-associated Protein 4
DAMPs: damage-associated molecular patterns
DDR: DNA damage response
DNA: deoxyribonucleic acid
DNA-PKcs: DNA-dependent protein kinase catalytic subunit
DOX: doxorubicin
DR5: death receptor 5
DSBs: Double-Strand Breaks
ER: estrogen receptor
ETAA1: Ewing tumor-associated antigen 1
FADD: FAS-associated death domain protein
FAS: Fas Cell Surface Death Receptor
FASL: FAS receptor ligand
FDA: Food and Drug Administration
FLICE: FADD-Homologous ICE/CED-3–like Protease
FLIP: FLICE/caspase-8 inhibitory protein
GATA4: GATA-binding factor 4
GC: guanine-cytosine pair
GLUT1: Glucose transporter 1
GPX4: Glutathione Peroxidase 4
GSDMD: Gasdermin D
H2AX: H2A histone family member X
H3K9: Histone H3 lysine 9
HDAC: Histone deacetylases
HDL: high-density lipoprotein
HER-2: human epidermal growth factor receptor 2
HR: homologous recombination
ICE: interleukin-1β–converting enzyme
IFN: interferon
IL: interleukin
IRE: iron-response elements
IRP: iron regulatory proteins
JNK: c-Jun N-terminal kinases
MAPKs: Mitogen-activated protein kinases
MCM2–7: minichromosome maintenance proteins 2–7
MDC1: Mediator of DNA Damage Checkpoint 1
MDR1: multidrug-resistance 1
MMP: matrix metalloproteinase
MPEG: methoxy-polyethylene-glycol
MPTP: mitochondrial permeability transition pore
MRE11: Meiotic recombination 11
MRN complex: Mre11, Rad50 and Nbs1 complex
mRNA: messenger ribonucleic acid
MRP1: Multidrug resistance-associated protein 1
Nbs1 (NBN): Nibrin protein, Nijmegen breakage syndrome
NFAT: Nuclear factor of activated T cells
NF-kB: Nuclear factor kappa-light-chain-enhancer of activated B cells
NHEJ: non-homologous end joining
NLR: NOD-like receptor
NLRP3: NLR family pyrin domain containing 3
NOXA: Phorbol-12-myristate-13-acetate-induced protein 1
NPLD: nonpegylated liposomal DOX
NRF-2: nuclear factor erythroid 2-related factor 2
PAMAM: Poly(amidoamine)
PARP1: Poly (ADP-ribose) polymerase 1
PCL-PEG: polycaprolactone-PEG
PD-1: Programmed cell death protein 1
PI3K: Phosphoinositide 3-kinases
PIKKs: Phosphatidylinositol 3-kinase-related kinases
PLD: Pegylated liposomal DOX
PLLA-PEG: Poly(L-lactide)-b-poly(ethylene glycol)
PNKP: polynucleotide kinase phosphatase
PNPs: Polymeric nanoparticles
PR: progesterone receptor
pRb: Retinoblastoma protein
PSP: Polysaccharopeptide
PTEN: Phosphatase and tensin homolog
PUFA: polyunsaturated fatty acid
PUMA: p53 upregulated modulator of apoptosis
RAAS: renin-angiotensin-aldosterone system
RHINO: Rad9, Rad1, Hus1 interacting nuclear orphan complex
RIP: receptor-interacting protein
ROS: reactive oxygen species
RPA, ATRIP: replication protein A/ATR-interacting protein
RPA: replication protein A
SiNPs: Silica nanoparticles
SLNs: Solid-lipid nanoparticles
SMAC (DIABLO): second mitochondria-derived activator of caspases
SOD2: superoxide dismutase 2
SSBs: Single-Stranded Breaks
TDP-1: tyrosyl-DNA phosphodiesterase 1
TNBC: triple-negative breast cancer
TNFR1: tumor necrosis factor receptor 1
TOPBP1: DNA Topoisomerase II Binding Protein 1
TP53: tumor protein p53
TRAIL: TNF-related apoptosis-inducing ligand
XR-CC1: X-ray repair cross-complementing group-1
